# Impact of Water Stress on Metabolic Intermediates and Regulators in Broccoli Sprouts, and Cellular Defense Potential of Their Extracts

**DOI:** 10.3390/ijms26020632

**Published:** 2025-01-13

**Authors:** Ivana Šola, Daria Gmižić, Karlo Miškec, Jutta Ludwig-Müller

**Affiliations:** 1 Department of Biology, Faculty of Science, University of Zagreb, Horvatovac 102a, 10000 Zagreb, Croatia; 2 Faculty of Biology, Technische Universität Dresden, Zellescher Weg 20b, 01217 Dresden, Germany

**Keywords:** Brassicaceae, glucosinolates, plant hormones, polyphenolics, proteins, sugars, vitamins

## Abstract

Drought and flood (water stress) alter plant metabolism, impacting the phytochemical content and biological effects. Using spectrophotometric, HPLC, and electrophoretic methods, we analyze the effects of water stress on broccoli (*Brassica oleracea* L. convar. *botrytis* (L.) Alef. var. *cymosa* Duch.) sprouts. Drought and flood differently influenced chlorophylls, carotenoids, and porphyrins, with drought having a stronger inhibitory effect on chlorophyll a, total chlorophyll, and porphyrins. Carotenoids and glucosinolates increased under drought but decreased with flooding, suggesting that these compounds play a crucial role in drought tolerance. Nitrate increased with drought from 13.11 ± 1.05 mg/g dw to 22.41 ± 1.20 mg/g dw but decreased under flooding to 5.17 ± 1.03 mg/g dw, and oxalic acid was reduced by drought only (from 48.94 ± 1.30 mg/g dw to 46.43 ± 0.64 mg/g dw). Flood reduced proteins by 29%, phenolics by 15%, flavonoids by 10%, flavonols by 11%, tannins by 36%, and proanthocyanidins by 19%, while drought decreased flavonoids by 23%. Total phenolics and proanthocyanidins were increased by drought by 29% and 7%, respectively, while flooding decreased hydroxycinnamic acids by 13%. Both stress types influenced individual polyphenols differently: drought diminished ferulic acid by 17% and increased sinapic acid by 30%, while flooding reversed these effects and enhanced kaempferol by 22%. These compounds, along with proline (which increased by 139% under drought), emerged as biomarkers of water stress. Flood impacted antioxidant capacity more significantly, while drought-stressed broccoli extracts better protected plasmid DNA against oxidative damage. These findings underline the metabolic plasticity of broccoli sprouts and their potential in targeted crop management for water stress resilience.

## 1. Introduction

Investigating the effects of drought and flooding on plant growth and yield production is highly relevant for understanding their resilience in a rapidly changing climate. The occurrence of extreme conditions is becoming more frequent due to climate change, with significant implications for agriculture, ecosystems, and global food security. Plants use different physiological, molecular, and biochemical mechanisms to cope with drought or flood stress. Recently, a theoretical framework, as a foundation for creating mathematical models, has been proposed that would capture and describe how plants respond to varying soil moisture levels, ranging from drought conditions to flooding [1].

Broccoli (*Brassica oleracea* L. convar. *botrytis* (L.) Alef. var. *cymosa* Duch.) sprouts are recognized for their high levels of bioactive compounds, including glucosinolates, phenolics, flavonoids, vitamins, and antioxidants, all of which, collectively, contribute to their health-promoting effects, such as anticancer, anti-inflammatory, and cardioprotective properties [2]. However, the concentration of these phytochemicals, and, consequently, the biological activities of extracts, are strongly influenced by environmental factors, particularly drought and flood stress [3,4]. These two forms of abiotic stress impose very different physiological demands on plants, triggering unique metabolic responses that alter the synthesis, degradation, and activity of key phytochemicals. Understanding how drought and flooding affect the phytochemical and nutrient profile, antioxidant capacity, and DNA-protective activity of broccoli sprouts is critical, given the importance of these sprouts as functional foods and their potential in supporting human health.

Drought stress leads to water scarcity, creating osmotic stress and an increase in reactive oxygen species (ROS), which can cause oxidative damage at the cellular level [5]. To counter this, plants activate multiple defense mechanisms, including the accumulation of specialized metabolites, which play key roles in mitigating ROS damage [5]. Drought has been shown to increase the synthesis of glucosinolates, particularly glucoraphanin, a precursor to sulforaphane. Sulforaphane is a potent inducer of detoxifying enzymes and has been linked to anticancer effects. Glucosinolates are known to be part of the defense response of plants against abiotic and biotic stress [6]. Therefore, its increased concentration could enhance the health benefits of broccoli sprouts, as higher glucoraphanin levels would potentially lead to greater sulforaphane bioavailability. Drought often leads to an accumulation of phenolic acids such as caffeic and ferulic acids [7]. These compounds have strong antioxidant properties, helping neutralize ROS generated under water-limited conditions. Phenolics not only scavenge free radicals, but also contribute to the structural integrity of plant cells under stress [8], and protect the DNA structure against the mutagenic and toxic effects of UV light and H_2_O_2_ [9,10,11]. Increased phenolic content can enhance the nutritional and medicinal properties of broccoli sprouts, as these compounds have been linked to anti-inflammatory and anticancer effects in humans. Drought stress tends to elevate levels of flavonoids such as quercetin and kaempferol [12], both of which are known to function as powerful antioxidants. These flavonoids help scavenge ROS and protect cells from oxidative stress. In addition, flavonoids play a role in UV protection [13], which may be relevant in drought-prone, high-light environments. This increase in flavonoid concentration may amplify the antioxidant potential of broccoli sprouts, making them more beneficial for human consumption under drought conditions. Key enzymes involved in the antioxidant response, including superoxide dismutase (SOD), catalase (CAT), and peroxidase (POD), are typically upregulated under drought stress [14,15]. SOD catalyzes the conversion of superoxide radicals to hydrogen peroxide, which is then detoxified by CAT and POD, effectively reducing oxidative damage. The enhanced activity of these enzymes not only protects plant tissues, but may also increase the sprouts’ overall antioxidant capacity, which is beneficial for the health when consumed. Phytohormones also contribute to drought resistance in plants [16]. Signal transduction and reactive oxygen clearance are key drought stress mechanisms mediated by phytohormones like abscisic acid (ABA), salicylic acid, jasmonic acid, and auxin. Using two transcriptomic methodologies, reference and de novo, a potential candidate gene for drought tolerance—the transcription factor bHLH112—in two drought-sensitive *B. oleracea* L. var. *botrytis* x *italica* Sicilian landraces and two drought-tolerant *B. macrocarpa* Guss populations has been identified [17]. Water shortage reflects on the concentration of vitamins in plants as well. For example, in leaves and stems of soybean plants grown under drought, ascorbic acid levels were reduced, and this was due to the negative effect on biosynthesis, likely unrelated to substrate limitation, suggesting transcriptional or post-transcriptional regulation [18].

Flood stress, in contrast, results from excess water, leading to hypoxic conditions (low oxygen) in the root zone which impair aerobic respiration and shift the plant to anaerobic metabolism [14]. This change affects the synthesis and activity of phytochemicals differently from drought, often limiting specialized metabolism and altering antioxidant responses. Under flooding, the synthesis of glucosinolates is often reduced to save the resources for other metabolites more relevant for adjustment to excess water stress [19]. Hypoxic conditions may inhibit pathways that synthesize these compounds, leading to lower concentrations in broccoli sprouts. This reduction could, potentially, diminish the anticancer benefits associated with glucoraphanin and its derivative sulforaphane, as less of this bioactive compound may be available for conversion. Conversely, flooding tends to increase the content of phenolics and flavonoids [20]. In combination with high temperatures, waterlogging upregulates the stress-responsive protein ribosome-binding factor A in broccolis [21].

This study aims to comprehensively evaluate how drought and flood stresses influence the phytochemical and nutrient profiles, physiological parameters, antioxidant capacity, and the ability of broccoli extracts to protect plasmid DNA structure from ROS. By specifically examining changes in compounds such as flavonoids, phenolic acids, proteins, soluble sugars, glucosinolates, vitamins, hormones, antioxidant capacity, and potential of broccoli extracts to protect the DNA structure from oxidative damage, this research seeks to clarify how contrasting water stress conditions affect the health-promoting qualities of broccoli sprouts. Our hypothesis is that drought and flooding will differentially affect the metabolism of broccolis, and certain metabolites might be used as biomarkers of shortage and excess of water. Insights gained from this work will help optimize cultivation practices, potentially enhancing the nutritional and medicinal benefits of broccoli sprouts, especially under varying environmental conditions influenced by climate change.

## 2. Results and Discussion

Water stress significantly impacts plant metabolism, altering the phytochemical content and biological effects. Key mechanisms include stress signal perception and stimulation of various stress signaling pathways such as calcium (Ca^2^⁺) signaling, the mifigutogen-activated protein kinase (MAPK) pathway, hormone signaling, and ROS signaling [22]. The concentration of cytoplasmic Ca^2^⁺, a universal second messenger in plant stress signaling, fluctuates in response to water stress and hormones [23]. These fluctuations transduce Ca^2+^ signals through calmodulin, i.e., calcium-dependent protein kinases, and calcineurin B-like proteins to downstream targets, i.e., calcium-sensor genes. Highly conserved MAPKs modify specific target molecules through phosphorylation, regulating the activity of phospholipases, proteins associated with microtubules and the cytoskeleton, other kinases, and various transcription factors [24]. The concentration of the hormone ABA increases under stress and serves as a signal for the plant cells to adapt [25]. Therefore, the receptors for ABA are found in different parts of the cell, cytosol, nucleus, plasma membrane, and chloroplast envelope [22]. It has been revealed that ABA is able to regulate even 10% of all signaling genes in *Arabidopsis thaliana* [26]. It also regulates the transcription of MAPK genes in plants [22]. Maintenance of balanced ROS levels under the stress is accomplished by enzymatic and non-enzymatic antioxidants in plants [22]. Enzymatic machinery includes superoxide dismutase, catalase, ascorbate peroxidase, dehydroascorbate reductase, glutathione reductase, guaiacol peroxidase, and monodehydroascorbate reductase. On the other hand, some of the non-enzymatic antioxidants recruited for ROS scavenging are α-tocopherol, flavonoids, reduced glutathione, ascorbic acid, carotenoids, and osmolyte proline. All this suggests that water stress drives a wide range of cellular responses. Such changes could enhance the accumulation of bioactive compounds in plants, potentially boosting their DNA protective properties. This study explores the interplay between water stress and the nutritional and functional attributes of broccoli sprouts. In the Supplementary Figure S1, the phenotypes of broccoli sprouts grown under control water regime, drought, and flooding are shown. Detailed variations in morphometric traits under stress, such as weight, hypocotyl length, root width, root weight, root length, and cotyledon area were not assessed; therefore, this is a limitation.

### 2.1. Effect of Drought and Flooding on the Photosynthetic Parameters of Broccoli Microgreens

Both drought and flooding reduced the amount of Chl *a*, and the effect of drought (a reduction of 18%) was more significant than the effect of flooding (a reduction of 12%) (Table 1). Chl *b* was also diminished by both types of water stress, and there was no significant difference between the effect of shortage and excess of water, a decrease of 25% and 22%, respectively. Obviously, water stress was more detrimental for Chl *b* than Chl *a* in broccoli sprouts. Total chlorophyll was lowered by 21% by drought, and by 17% by flooding. Similar results regarding drought have been recorded in *B. oleracea* L. [27], white clover (*Trifolium repens*) [28], cotton [29], and quinoa [30]. The Chl *a*/*b* was 1.81 in the control plants, not significantly affected by drought, but increased to 2.05 under flooding. The fact that drought did not significantly alter the Chl *a*/*b* ratio indicates that the relative proportions of the reaction centers and light-harvesting complexes were maintained. This stability of the photosynthetic pigments suggests that drought might not have caused severe damage to the photosynthetic machinery, or that plants activated protective mechanisms to preserve the photosystem’s function. However, the increase in the Chl *a*/*b* ratio under flood stress suggests a reduction in Chl *b* relative to Chl *a*. Flood-induced hypoxia could disrupt the synthesis of Chl *b*, leading to a decline in light-harvesting complexes. Carotenoids were notably enhanced by drought (from 0.73 ± 0.03 mg/g dw in control plants to 0.80 ± 0.03 mg/g dw). On the contrary, flooding significantly reduced their amount (0.61 ± 0.02 mg/g dw). Total pigments, chlorophylls, and carotenoids, together, were reduced by both drought and flooding, with no significant difference between stress types. Furthermore, total porphyrins were also reduced, but the effect of drought was significantly stronger than the effect of flooding (a decrease of 22% and 15%, respectively). In general, drought showed a stronger negative effect than flooding on Chl *a*, total chlorophyll, and total porphyrins. A photosynthetic parameter that was more susceptible to flooding than to drought was total carotenoid content. Similar results had already been recorded for young Chinese cabbage [3].

### 2.2. Effect of Drought and Flooding on Sugars in Broccoli Microgreens

**Figure 1:**
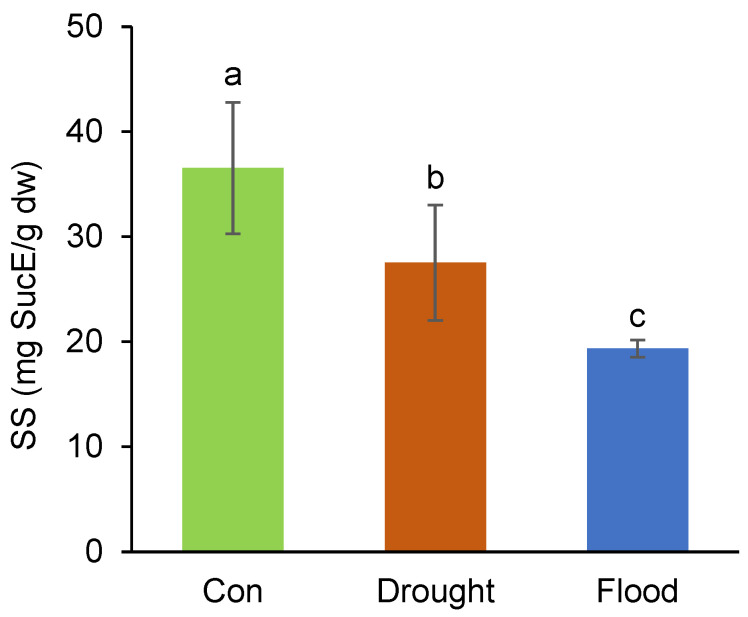
Effects of different watering regimes on soluble sugars in broccoli sprouts. Values represent the average ± standard deviation of three biological and three technical replicates. Different letters indicate a significant difference among the values (one-way ANOVA, Duncan’s test, *p* ≤ 0.05). dw = dry weight; SucE = sucrose equivalent; SS = total soluble sugars.

Drought stress often leads to an increase in soluble sugars in many plants, as these sugars act as osmoprotectants, helping stabilize cell structures, protect cellular proteins, and reduce water loss [31]. However, under stressful conditions, the metabolism of soluble sugars is a dynamic process that involves both breakdown and synthesis reactions happening at the same time [32], a phenomenon which can result in the reduction of total sugars as well. For example, sucrose and glucose serve as substrates for cellular respiration or act as osmolytes to help maintain cell balance, while fructose does not contribute to osmoprotection and appears to be involved in the production of specialized metabolites [32,33]. In our study, both types of stressors significantly lowered the number of soluble sugars in broccoli sprouts, with flooding being more detrimental than drought (a reduction of 47% and 25%, respectively) (Figure 1). As for the latter response, this could have happened because drought stress often limits photosynthesis due to stomatal closure, which reduces the CO_2_ intake. Lower photosynthesis rates reduce the production of carbohydrates, leading to lower soluble sugar levels. Severe drought can also damage cellular structures, including chloroplasts, further reducing the plant’s ability to produce and accumulate sugars [34]. However, flooding generally decreases soluble sugar content because waterlogged conditions reduce oxygen availability in the soil, leading to hypoxia or anoxia in the roots [35,36]. Under low oxygen conditions, aerobic respiration is limited, and plants switch to the less efficient anaerobic respiration, which generates much less energy and may deplete sugar reserves. Similar to drought stress, flooding can lead to chlorosis and reduced chlorophyll content, limiting photosynthesis and sugar production. A reduction in chlorophyll occurred in the broccoli sprouts as well (Table 1). Plants under flood stress also need more energy to cope with anaerobic conditions, which can quickly deplete sugar reserves.

### 2.3. Effect of Drought and Flooding on Nitrates and Proteins in Broccoli Microgreens

Nitrate (NO_3_^−^) functions as a source of an essential element integral to a wide range of macromolecules and acts as a signaling molecule that influences global gene expression and shapes the architecture of both root and shoot systems [37]. Nitrate was extremely affected by both types of stress. Drought increased its content up to even 71%, while flooding decreased the content by 61% (Table 2). Drought stress slows down various metabolic processes in plants, including nitrogen assimilation. This means that, even if some nitrate is absorbed by plants, it might not be efficiently converted into organic nitrogen forms (like amino acids) due to the stress-induced slowdown in nitrate reductase activity [38], further contributing to nitrate accumulation. Furthermore, flooding leads to waterlogged conditions, where the soil becomes anaerobic and, consequently, the microbial community is altered, a phenomenon which can lead to changes in their capacity to metabolize nitrogen in different forms. Flooding can also lead to leaching, a process by which soluble nutrients like nitrate are washed away from the root zone. Since nitrate is highly soluble in water, excess water from flooding can carry nitrate deeper into the soil profile or into the groundwater, effectively removing it from the reach of plant roots. This contributes to the decrease in nitrate availability in the topsoil. Similar results to ours were detected in taro (*Colocasia esculenta* cv. Daikichi) [39].

In broccoli sprouts, flooding significantly reduced the amount of total proteins (from 36.93 ± 1.83 mg BSAE/g dw to 26.16 ± 2.32 mg BSAE/g dw), while drought did not show a significant impact on the same parameter (Table 2). The contrasting effects of drought and flooding on protein content underscore the plant’s ability to tailor its metabolic responses according to the specific type of stress. Drought appears to induce adaptive mechanisms that protect protein synthesis, while flooding seems to hinder it due to oxygen limitations. This distinction implies that broccoli sprouts may have evolved stress-specific pathways that favor protein stability under drought, while protein synthesis is compromised under flooding due to the prioritization of immediate survival processes. For growers, these data reinforce the importance of well-drained soils for optimal broccoli sprout protein yield, as flooding not only reduces protein content, but might also compromise plant health and overall growth.

### 2.4. Effect of Drought and Flooding on the Phytochemical Content of Broccoli Microgreens

Total glucosinolates were differently affected by drought and flooding (Table 3). While a shortage of water increased their content by 44%, excessive water was detrimental and lowered their amount by 12%. Similar results regarding drought conditions have been recorded in Chinese cabbage (*B. rapa* ssp. *pekinensis*) as well [40,41], *B. oleracea* L. var. *capitata* [42,43], and *B. rapa* ssp. *rapifera* L. [44]. On one hand, in broccolis (*B. oleracea* L.) [45] and *A. thaliana* [46], drought has been found to decrease glucosinolates. On the other hand, no significant changes in oilseed rape (*B. napus*) in total glucosinolates regarding the flood effect have been reported [47]. The contrasting effects of drought and flooding on glucosinolate levels suggest that these compounds are more crucial for drought tolerance than for flood resilience. Under drought conditions, glucosinolates could potentially contribute to osmotic stress tolerance indirectly or in specific contexts. For example, glucosinolates are linked to hormonal signaling pathways that overlap with stress response mechanisms [48]. This crosstalk might help plants fine-tune their reactions to osmotic stress or contribute to stabilizing cellular structures, whereas, under flooding, the plant might prioritize other mechanisms, like aerenchyma formation or anaerobic respiration, to adapt to waterlogged conditions. This implies that glucosinolate pathways may be selectively activated based on the type of stress, showing that plants dynamically adjust their metabolic priorities depending on environmental demands. Together, these observations highlight that plants respond differently to water stress, and the direction of glucosinolate adjustment (increase or decrease) is closely linked to the type and severity of water imbalance. This variability underscores the complexity of plant stress responses and suggests that glucosinolate accumulation may be a strategic adaptation that plants deploy selectively based on environmental conditions.

Oxalic acid, a well-known calcium-bioavailability regulator, serves in herbivory defense mechanisms, is a chelator of heavy metals, and helps nutrient acquisition from soil and pH balance [49]. In our study, oxalic acid was affected by water shortage and it was reduced by 5% (Table 3). This is contrary to the results on wheat (*Triticum aestivum*) [50]. Excess of water showed no significant effect. Since oxalic acid plays a role in regulating calcium and detoxifying metals, it is possible that, under mild, room-temperature drought stress, the plant’s overall metabolic activity slows down, reducing the need for oxalic acid production and causing a reduction in its levels. On the other hand, drought induces osmotic stress in plants, forcing them to prioritize the synthesis of osmolytes (like proline) to maintain cellular water balance. This prioritization may divert resources away from oxalic acid production, leading to a decrease in its levels.

Total phenolics were enhanced by drought by a notable 29% (Table 3). Similar results have been recorded in different genotypes of *B. oleracea* L. [27]. However, flooding lowered their amount significantly (−15%). This was similar to the case of total glucosinolates. The contrasting effects of drought and flooding on total phenolics suggest that the response of these compounds in broccolis varies significantly depending on the level of water in the soil. The opposite effects were recorded for total proanthocyanidins as well, as drought increased them by 7%, while flooding reduced them by 19%.

Total flavonoids were reduced by each stress type, with drought showing a significantly stronger effect than flooding (a reduction of 23% and 10%, respectively). Total phenolic acids were not substantially affected, while total hydroxycinnamic acids were decreased by excessive water. Similarly, total flavonols and tannins were reduced by the excess of water by 11% and 36%, respectively.

Soil moisture levels have a significant impact on the concentrations of key phenolic compounds in plants, with notable differences between the effects of excess water and drought conditions (Table 4). Among the identified individual phenolic components in broccoli sprouts, the highest concentration was recorded for sinapic acid, 2.04 ± 0.03 mg/g dw, and the flavonol kaempferol, 0.71 ± 0.03 mg/g dw. Excessive water in the soil significantly increased the concentration of ferulic acid (56% enhancement); however, it reduced the concentration of sinapic acid (a 13% decrease). Drought also reduced ferulic acid (a 17% reduction), but increased sinapic acid by about 30%.

Kaempferol was affected in a similar direction as ferulic acid, since drought notably reduced its concentration (−18%), while flooding enhanced it (22%). Another flavonol, quercetin, was not affected by water shortage, but was considerably elevated by the excess of water (38%). This suggests that these compounds may be synthesized or accumulated in response to water stress caused by flooding, potentially as part of a protective mechanism in plants to counteract the adverse effects of excessive moisture. The higher concentration of these compounds under waterlogged conditions could be linked to changes in plant metabolism that favor antioxidant accumulation to mitigate oxidative stress induced by flooding. Conversely, drought stress resulted in a substantial increase in sinapic acid concentrations, highlighting a differentiated metabolic response where certain polyphenolics, like sinapic acid, may be preferentially synthesized to counter drought-induced stress. The differential responses of these polyphenolic compounds to water availability indicate that plants modulate their metabolic pathways according to the specific stressor. While ferulic acid, kaempferol, and quercetin appear to respond positively to excess water, sinapic acid synthesis or accumulation is more prominent under drought conditions.

In conclusion, the study underscores that water stress—whether an excess or a deficit—induces specific shifts in polyphenolic compound concentrations, reflecting a complex and compound-specific adaptive strategy in plants. The pronounced changes in ferulic acid, kaempferol, and quercetin under excess water conditions, and in sinapic acid under drought, may indicate their roles in plant resilience under varying environmental stresses. Understanding these metabolic adjustments can inform agricultural practices aimed at enhancing stress tolerance in crops.

Based on the results, sinapic and ferulic acid and the flavonoid kaempferol show potential as biomarkers for identifying and quantifying drought and flood stress in plants due to their distinct and consistent responses to varying water conditions. By analyzing the concentrations of these polyphenolic compounds, it would be possible to distinguish between drought and flooding conditions, helping early diagnosis and the development of targeted crop management strategies for specific water stresses.

### 2.5. Effect of Drought and Flooding on the Vitamins in Broccoli Microgreens

The data in Table 5 show a minor trend where drought had a slightly greater effect on the induction of *L*-ascorbic acid and flooding appeared somewhat more influential on increasing folic acid. However, neither drought nor flooding significantly affected the concentration of *L*-ascorbic (vitamin C) and folic acids in broccoli sprouts, leading to the conclusion that the concentrations of *L*-ascorbic and folic acids in broccoli sprouts remain largely stable under both drought and flood stress conditions. This stability may indicate that *L*-ascorbic and folic acid pathways in broccoli sprouts are either less sensitive to water stress or actively regulated to maintain consistent levels under varying environmental conditions. For comparison, Ben Ammar et al. [27] detected different behaviors of different broccoli genotypes. Namely, three genotypes (CI5, CI6, and CV2) significantly reduced the level of *L*-ascorbic acid under drought stress, while genotypes BR3, BR4, and CV3 greatly increased their vitamin C content following drought stress. For *L*-ascorbic acid, this could be linked to its critical role as an antioxidant; maintaining stable levels may help ensure adequate protection against stress-induced oxidative damage regardless of moisture conditions. Similarly, stable folic acid levels could be essential for sustaining key metabolic processes, including DNA synthesis and repair, even under suboptimal water conditions. In summary, the resilience of *L*-ascorbic and folic acid concentrations in broccoli sprouts under both drought and flooding conditions suggests that these vitamins may not be reliable indicators of water stress. Instead, their consistent levels may reflect a protective or homeostatic response in broccoli sprouts, ensuring that essential metabolic and antioxidant functions are maintained despite environmental challenges.

### 2.6. Effect of Drought and Flooding on the Oxidative Stress Parameters in Broccoli Microgreens

Each water stress type substantially altered H_2_O_2_ concentrations and, in an opposite way, drought increased the level of H_2_O_2_ (26%), while flooding decreased it (−21%) (Table 6). Drought stress is known to trigger an increase in H_2_O_2_ levels in plants [17,51]. Drought causes a variety of physiological stresses in plants, including dehydration, stomatal closure, and impaired photosynthesis. These stress conditions disrupt the balance between the production and detoxification of ROS, such as H_2_O_2_, leading to an overall increase in ROS levels [52]. Flooding, however, typically leads to hypoxia or even anoxia in the root zone due to water saturation. Since oxygen is required for the normal generation of ROS, including H_2_O_2_, reduced oxygen availability under flooding conditions limits the production of ROS.

The amino acid proline is a stress adaptor molecule known to enhance biotic/abiotic tolerance responses in plants [53]. Its primary role is to help them adjust to osmotic stress. It has been shown that, under salt stress, drought, UV radiation, heavy metal ions, pathogens, and oxidative stress, proline tends to accumulate in plants [53]. In our study, there was also an enormous increase (139%) in proline under the effect of water shortage. In contrast, no effect was recorded in response to flooding. Similar results have been recorded in different wheat (*T. aestivum*) genotypes [50].

The level of malondialdehyde (MDA), a key indicator of lipid peroxidation and, thus, oxidative stress, was markedly changed under drought and flooding. Interestingly, drought decreased the level of MDA in broccoli plants, flooding increased it by 26%. This is somewhat unexpected, because drought is generally associated with increased oxidative stress, which would typically lead to higher levels of MDA. One potential explanation could be that the broccoli microgreens have efficient antioxidant defenses (such as higher levels of proline or antioxidant enzymes) that mitigate oxidative damage, leading to lower MDA accumulation despite the drought stress. Indeed, we recorded a substantial increase in proline in broccolis grown under drought stress (Table 6). A similar correlation between proline accumulation and oxidative damage of cellular lipids was detected by Natarajan et al. [54] as well. In contrast to drought, flooding led to a notable increase in MDA levels. This is consistent with what would be expected under flooding conditions, as flooding creates hypoxic or anoxic conditions in the roots which can induce oxidative stress once the oxygen supply is restored [55]. This process can lead to a burst of ROS and subsequent lipid peroxidation, raising MDA levels. Flooding also disrupts normal cellular metabolism and weakens cell membranes, contributing to increased MDA as a marker of lipid peroxidation [55]. Similar results have been reported for adzuki bean (*V. angularis*) [56].

### 2.7. Effect of Drought and Flooding on the Antioxidant Capacity of Broccoli Microgreens

The antioxidant capacity of broccoli sprout extracts was determined with three different methods (Table 7). Drought enhanced the antioxidant capacity of broccoli sprouts measured with ABTS and DPPH as substrates by 9% and 16%, respectively. Even though the level of increase was relatively modest, antioxidants often function in a cumulative and synergistic manner [57]. This suggests that drought triggers an adaptive response in the plants, leading to the accumulation of antioxidant compounds, particularly those with a hydrogen-donating capacity. This response is likely a protective mechanism to scavenge excess ROS generated under water-deficient conditions. The increase in antioxidant capacity under drought conditions indicates that broccoli sprouts might have an enhanced nutritional value when grown in water-limited environments, as antioxidant compounds are beneficial for human health. This insight could be valuable for agricultural practices and nutritional research, as controlled drought conditions might be used strategically to enhance the antioxidant profile of sprouts and, potentially, other crops.

Under flooding stress, the results do not seem to be as consistent for the three methods. While, with ABTS, the antioxidant capacity seemed to be reduced by 18%, when using DPPH as the scavenging method the capacity increased by 43%. Such opposite results between the ABTS and the DPPH assay reflect the complex response of the broccolis’ antioxidant system to flooding, where specific antioxidant compounds fluctuate differently. This suggests that flooding alters specific components of the antioxidant profile rather than uniformly increasing or decreasing the overall antioxidant capacity. For a comprehensive assessment, it may be necessary to analyze the specific compounds contributing to each assay to better understand the nuanced antioxidant response under flooding conditions.

Flooding caused a higher shift in antioxidant capacity of broccoli sprouts than drought. This suggests that broccoli plants experience a more intense oxidative stress response under excess water conditions. Flooding creates an environment where oxygen availability in the root zone is drastically reduced due to water saturation. This lack of oxygen, or hypoxia, can disrupt cellular respiration and lead to the accumulation of ROS which, in turn, triggers a strong antioxidant response. The larger shift in antioxidant capacity under flooding compared to drought may indicate that broccoli sprouts produce or activate specific antioxidants in response to the intense stress of excess water. Since we found that excess water notably enhanced ferulic acid, kaempferol, and quercetin (Table 4), we assume that these polyphenolics might be among the crucial mediators of stress tolerance in response to low oxygen availability. From an agricultural perspective, understanding these responses can provide insights into optimizing growth conditions to enhance antioxidant properties in crops. In practical terms, flooding conditions could be managed to boost certain antioxidant compounds, although excess flooding may also negatively impact overall plant health and yield.

The antioxidant potential measured by the ABTS method, very strongly, according to Evans [58], positively correlated with total carotenoids, phenolics, tannins, proanthocyanidins, sinapic acid, and the hormone IAA (Supplementary Table S1). The antioxidant potential measured with DPPH revealed very strong positive correlations with Chl *a*/*b* ratio and salicylic acid.

### 2.8. Effect of Drought and Flooding on the Hormones in Broccoli Microgreens

Auxin, indole-3-acetic acid (IAA), is a key plant hormone that significantly promotes growth [59] and plays an integral part in plant adaptation to drought stress [60]. Flooding lowered the concentration of IAA by 78%, while water shortage did not show an effect (Table 8). Similar results have been recorded in *Cleistocalyx operculatus* and *Syzygium jambos* leaves due to soil waterlogged conditions [61], in the ‘Hayward’ cultivar of kiwifruit [62], and in the needles of several *Pinus radiata* cultivars [63]. In rice (*Oryza sativa* subsp. *japonica*), a decreased IAA content has also been observed [64]. IAA is primarily synthesized from tryptophan through pathways like the indole-3-pyruvic acid (IPA) pathway [65]. This process relies on aerobic conditions for certain enzymatic reactions (e.g., TAA1 and YUCCA enzymes). After a certain time of flooding, reduced oxygen levels hinder these reactions, leading to decreased IAA production [66]. Additionally, flooding triggers the synthesis of ethylene [67], which acts as a signaling molecule that can downregulate genes responsible for IAA biosynthesis and promote IAA degradation by activating enzymes such as IAA oxidase, which catalyzes the oxidation of IAA into inactive forms.

Abscisic acid is a plant hormone often associated with abiotic stress factors. Due to the high standard deviations for ABA levels, the results were difficult to interpret. Nevertheless, a tendency of decrease under both water stress types could be noticed. Similar result under flooding conditions have already been recorded in the adventitious roots of wheat (*T. aestivum*) [68], in deepwater rice [69], and in *Rumex palustris* [70]. The trend showing a reduction in ABA under drought stress was rather unexpected, since ABA is more often increased in such conditions [71]. However, our data and the results of Privitera et al. [17], who detected two downregulated genes, Bo4g190030 and Bo7g075740, correlated with ABA signaling pathways in drought-sensitive *B. oleracea* L. var. *botrytis* x *italica* Sicilian landraces and drought-tolerant *B. macrocarpa* Guss populations, indicate specificity in ABA signaling during water scarcity conditions.

Salicylic acid was markedly increased under flooding (an 85% enhancement) and not affected by drought. Similar results have been recorded in the ‘Hayward’ cultivar of kiwifruit [62] and in waterlogged adzuki beans (*V. angularis*) [56]. The increase in salicylic acid might trigger defense mechanisms against potential pathogens that thrive in waterlogged conditions, enhancing the plant’s ability to cope with both biotic and abiotic stresses. It has also been shown that salicylic acid can trigger directly aerenchyma formation, which is one important functional hallmark of waterlogging [72].

### 2.9. Effect of Drought and Flooding on the Concentration of Genomic DNA in Broccoli Microgreens

**Figure 2:**
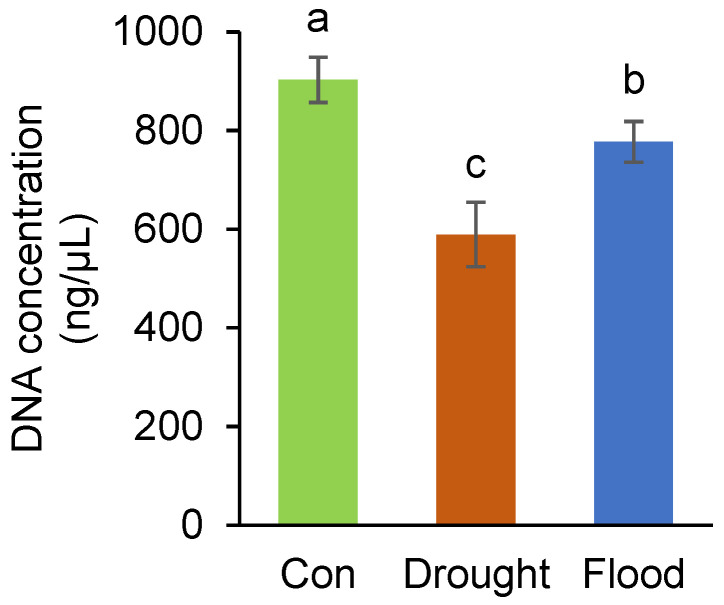
Effect of drought and flooding on the concentration of genomic DNA in broccoli microgreens. Values represent the average ± standard deviation of four biological replicates. Different letters indicate a significant difference among the values (one-way ANOVA, Duncan’s test, *p* ≤ 0.05).

In the control broccoli microgreens, the genomic DNA concentration was measured to be 902.95 ± 45.97 ng/µL (Figure 2). Both flooding and drought stress significantly affected the genomic DNA concentration. Under flooding conditions, the concentration decreased to 777.20 ± 41.50 ng/µL, while drought stress led to an even greater reduction, with a concentration of 589.48 ± 65.49 ng/µL.

The reduction in genomic DNA concentrations under both drought and flooding stress could be attributed to distinct physiological and biochemical impacts of water imbalances. During drought conditions, water scarcity leads to cellular dehydration, causing chromatin condensation and a reduction in the effective size of the nucleus [73]. This may impair DNA replication processes and reduce the availability of DNA for extraction, as observed in the significant decrease to 589.48 ± 65.49 ng/µL. Additionally, drought-induced oxidative stress generates ROS, which can directly damage DNA through strand breaks, base oxidation, and crosslinking [5], further contributing to the observed decrease. In contrast, excess water during flooding creates hypoxic conditions that disrupt mitochondrial respiration, leading to energy shortages and reduced cellular activity [74]. This might hinder the replication machinery and DNA synthesis, resulting in a concentration decrease to 777.20 ± 41.50 ng/µL. While both stressors significantly lowered DNA concentrations, the more severe impact of drought highlights its greater potential to disrupt genomic integrity and cellular metabolism.

### 2.10. Effect of Drought and Flooding on the Potential of Broccoli Microgreen Extracts to Protect Plasmid DNA Structure

**Figure 3:**
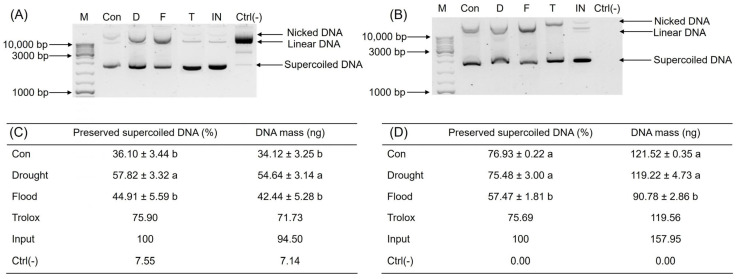
Effect of drought and flooding on the potential of broccoli microgreen extracts to protect plasmid supercoiled DNA from damage caused by (**A**) Fenton’s reagent and (**B**) UV/H_2_O_2_ photolysis, expressed relative to the concentration of the supercoiled DNA of an intact plasmid. (**A**,**B**) show the 1.5% agarose gel electrophoresis with three distinct plasmid conformations (supercoiled, linear, and nicked). The level of the supercoiled conformation preservation is shown after exposure to (**C**) the Fenton’s reagent and (**D**) UV/H_2_O_2_ photolysis relative to the intact plasmid, expressed in percentage, alongisde the corresponding DNA mass calculated from the standard curve, expressed in nanograms. A band densitometry was performed using the ImageJ (Fiji) software, version 2.2.0. Values represent the average ± standard deviation of three biological replicates. Different letters indicate a significant difference among the values in a row (one-way ANOVA, Duncan’s test, *p* ≤ 0.05). D = drought; F = flooding. M = GeneRuler DNA Ladder Mix (Thermo Fisher Scientific, USA); bp = base pair; Con = plasmid + control plants extract; F = plasmid + extract of flood-stressed plants; D = plasmid + extract of drought-stressed plants; T = plasmid + trolox 30 mg/mL; IN = intact plasmid; Ctrl(-) = plasmid + (**C**) Fenton’s reagent or (**D**) UV/H_2_O_2_; ng = nanogram.

The electrophoretic motif of plasmid DNA (pSgM1_HNF1A) was used as a model to test whether and, if yes, how drought and flooding treatments of broccoli microgreens affected its possibility to preserve supercoiled DNA forms exposed to the Fenton’s reagent (which generates highly reactive hydroxyl radicals that cause severe oxidative damage to the DNA, including strand breaks) or combined UV/H_2_O_2_. Such a damage can be determined by analyzing banding patterns of plasmid DNA separated using gel electrophoresis. Namely, plasmid DNA can exist in multiple conformations (supercoiled, nicked/relaxed circular, and linear), each of which migrates differently in an agarose gel. The intensity of each band reflects the amount of DNA present. A degree of DNA damage was assessed by evaluating changes in band intensity and pattern using the imaging software ImageJ, version 2.2.0 [75]. Drought-stressed broccoli extract was significantly more effective in the preservation of plasmid DNA exposed to the Fenton’s reagent (57.82 ± 3.32%) than control and flood-stressed broccoli extracts (36.10 ± 3.44 and 44.91 ± 5.59%, respectively) (Figure 3A,C), but on a level that was not significant. None of the extracts was as good in supercoiled DNA preservation as the standard Trolox, a well-characterized reference to evaluate the effectiveness of the extracts in preserving the supercoiled DNA structure, at a concentration of 30 mg/mL (75.90% of plasmid supercoiled DNA maintained). A second method to mediate damage to DNA is a treatment with UV radiations and H_2_O_2_. In this experiment, control and drought-stressed broccoli extracts were significantly more effective in the preservation of the supercoiled DNA form (76.93 ± 0.22% and 75.48 ± 3.00%, respectively) than flood-stressed broccoli extracts (57.47 ± 1.81%) (Figure 3B,D). In contrast to the treatments of the plasmid DNA with the Fenton’s reagent, extracts from UV/H_2_O_2_-treated plants were as effective in their protective role as Trolox. In general, broccoli extracts were more effective in the preservation of the supercoiled DNA from UV/H_2_O_2_ photolysis than from the Fenton’s reagent. Also, in both cases, extracts of drought-stressed broccolis were more effective than extracts of flood-stressed broccolis, and, in the case of the Fenton’s reagent, drought-stressed broccolis were even better than control plants in protecting the supercoiled DNA structure.

This suggests that drought-stressed broccoli extracts might offer greater protection against DNA degradation compared to control and flood-stressed extracts. The observed differences might be attributed to variations in phytochemical composition caused by environmental stress. Precisely, in broccolis grown under the drought effect, carotenoids (Table 1), glucosinolates, total phenolics, proanthocyanidins (Table 3), and sinapic acid (Table 4) were notably increased. Therefore, we assume that these compounds might be involved in DNA structure protection. The variability in DNA preservation across treatments highlights the need for further exploration of the specific compounds responsible for the effects.

The difference in results from the DNA protection experiments using the Fenton’s reagent and UV/H_2_O_2_ photolysis might be due to the distinct mechanisms of DNA damage and repair involved in each process. UV radiation directly interacts with DNA, leading to lesions that distort the DNA double helix, and the efficiency of DNA protection depends on how well the agent absorbs or dissipates UV energy before it interacts with the DNA [76]. The Fenton’s reagent, on the other hand, produces hydroxyl radicals that are highly reactive and cause oxidative damage to DNA, leading to single-strand breaks, double-strand breaks, and oxidized bases [77]. In terms of UV protection, phytochemicals generally work by blocking UV light or by dissipating the absorbed energy, while, in the case of the Fenton’s reagent, protective compounds scavenge free radicals to prevent the formation of hydroxyl radicals.

### 2.11. Chemometric Analyses

**Figure 4:**
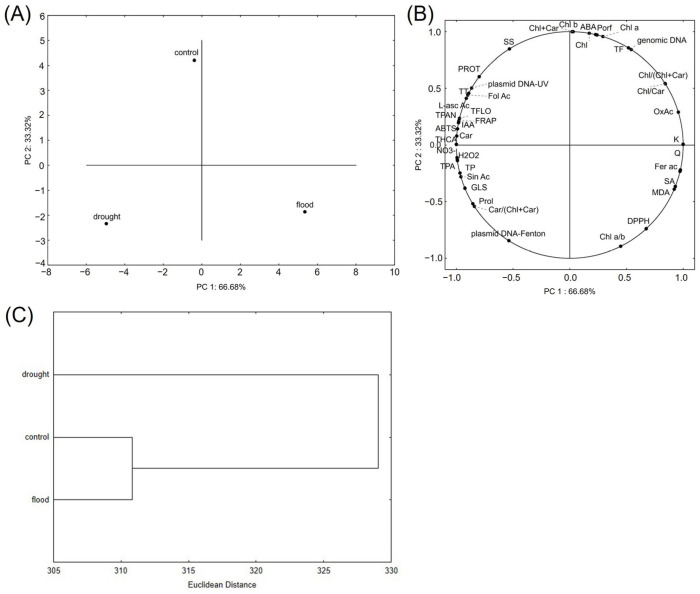
The principal component analysis showing (**A**) the relation among broccoli microgreens grown under control, flooding, and drought conditions based on the analyzed variables, whose grouping is shown in the (**B**) part of the figure. (**C**) Hierarchical clustering of broccoli microgreens grown under control, flooding, and drought conditions, expressed as the Euclidean distance based on the measured total and individual bioactive compounds, their pigments, hormones, antioxidant capacity, and their potential to protect the supercoiled DNA structure. ABA = abscisic acid; Car = carotenoids; Chl = chlorophyll; Fer Ac = ferulic acid; Fol Ac = folic acid; GLS = total intact glucosinolates; H_2_O_2_ = hydrogen peroxide; *L*-asc Ac = *L*-ascorbic acid; IAA = indole-3-acetic acid; K = kaempferol; NO_3_^−^ = total nitrates; MDA = lipid peroxidation; OxAc = oxalic acid; Porf = porphyrins; PROT = total proteins; Q = quercetin; SA = salicylic acid; Sin Ac = sinapic acid; SS = total soluble sugars; TF = total flavonoids; TFlo = total flavonols; THCA = total hydroxycinnamic acids; TP = total phenolics; TPA = total phenolic acids; TPAN = total proanthocyanidins; TT = total tannins.

Principal component analysis (PCA) showed clear separation of control and plants grown under drought and flood conditions (Figure 4A). Based on the PC 1, group grown under flood slightly more differed from control than the group grown under drought did. However, based on the PC2, drought slightly more affected plants than flood did. Parameters that contributed most to the separation of flood-group were lipid peroxidation expressed in MDA content, salicylic acid concentration, and antioxidant capacity measured by DPPH method (Figure 4B). Drought-grown plants were mostly separated due to the concentration of glucosinolates, proline and potential to protect DNA structure from Fenton’s reagent. According to hierarchical clustering, broccoli grown under drought was more distant from control group than those grown under flood conditions (Figure 4C).

When we performed the Pearson’s correlation analysis, the results revealed very strong or strong positive correlations between plasmid DNA protection against UV and total carotenoids, proteins, phenolics, tannins, proanthocyanidins, sinapic acid, hormone IAA, and ABTS results (Supplementary Table S1). In addition, a strong positive correlation of plasmid DNA protection against the Fenton’s reagent was detected with total glucosinolates, phenolics, and sinapic acid. Sinapic acid has already been recognized as a protecting compound against DNA damage [78,79,80]. On the other hand, correlations between genomic DNA concentrations and bioactive compounds were lower. We assume that this is because water stress primarily affects metabolic processes, such as photosynthesis, respiration, and the synthesis of stress-related proteins and metabolites, rather than genomic DNA integrity or quantity. Cellular responses to water stress involve transcriptional regulation rather than the alteration of the DNA content. For example, as already discussed at the beginning of this chapter, signaling pathways (e.g., MAPK, Ca^2^⁺ signaling) activate stress-responsive genes without necessarily altering the amount of genomic DNA. In addition, under water stress, secondary effects such as oxidative stress can lead to DNA damage (e.g., strand breaks or mutations) rather than changes in DNA concentrations.

## 3. Materials and Methods

### 3.1. Plant Material

Seeds of the broccoli breeding line *B. oleracea* L. convar. *botrytis* (L.) Alef. var. *cymosa* Duch., known as Ramoso Calabrese broccolis, Art. No. 424430, were obtained from International Seeds Processing (ISP) GmbH (Quedlinburg, Germany; https://www.isp-quedlinburg.de/english, accessed on 15 March 2024). This variety was chosen because it is among the most commonly used in human nutrition. The seeds were sterilized with 2.55% Izosan^®^ G (Pliva, Zagreb, Croatia) and the plants were grown on a sterile soil substrate Stender AG (Schermbeck, Germany) for 7 days in a climate chamber FitoClima 600PLH (Aralab, Rio de Mouro, Portugal) under regular conditions (23 °C day/18 °C night, 65% humidity), and then the treatment with drought (plants were watered every third day with a minimal volume of water) and flooding (plants were watered every day with an excessive volume of water and the pots were immersed in water) followed for the next 7 days. The control group was watered every day with a regular volume of water. After that, we collected the aerial part of the plants which were immediately frozen in liquid nitrogen and lyophilized in the Alpha 1–2 LSCbasic freeze-dryer (Martin Christ Gefriertrocknungsanlagen GmbH, Osterode am Harz, Germany). Lyophilized plants were homogenized into a powder and extracts were prepared in different solvents, depending on the analysis method. The material included three biological replicates (three separate groups grown under the same conditions) and three technical replicates which were weighed from each biological replicate.

### 3.2. Determination of Photosynthetic Pigments

The concentration of pigments was measured according to the method of Sumanta et al. [81]. The extract was prepared in 80% acetone at a concentration of 5 mg/mL. The absorbances were measured on a spectrophotometer (Thermo Scientific Nanodrop 2000c, Thermo Fisher Scientific, Waltham, MA, USA) at wavelengths of 470, 575, 590, 628, 645, and 663 nm. The concentrations of Chl *a* and *b* were determined according to Lichtenthaler (1987) [82], and the following equations were used:

$$Chl\;a=12.25\;Abs_{663}-2.79\;Abs_{645}$$

$$Chl\;b=21.50\;Abs_{645}-5.10\;Abs_{663}$$

where Abs = absorbance.

### 3.3. Determination of Soluble Sugars

Soluble sugars were determined as in our previous work [83]. Absorbance was measured at a wavelength of 485 nm. A sucrose solution in the range of 0.01–0.7 mg/mL was used for the calibration curve. The results are presented as milligrams of sucrose equivalents per gram of dry weight (mg SucE/g dw).

### 3.4. Determination of Nitrogen-Containing Bioactives

Nitrates were extracted with 70% (*v*/*v*) ethanol and measured at a wavelength of 405 nm, according to Hachya i Okamoto [84]. An aqueous solution of potassium nitrate in the range of 0.03125–2.0 mg/mL was used as a standard. The results are presented as milligrams per gram of dry weight (mg/g dw) of the sample. For the determination of total proteins, extracts were prepared in a potassium–phosphate buffer (100 mM), pH 7.0, containing 0.1 mM of EDTA and the absorbance was measured at a wavelength of 595 nm [85]. A solution of bovine serum albumine (BSA) in the range of 0.0625–0.5 mg/mL in the aforementioned buffer was used as a standard. The results are presented as milligrams of BSA equivalents per gram of dry weight (mg BSAE/g dw) of the sample.

### 3.5. Determination of Phytochemicals

Extracts of total intact glucosinolates were prepared in hot 70% methanol and the extraction procedure has already been described [86]. The absorbance was measured at a wavelength of 405 nm. An aqueous solution of sinigrin in the range of 0.1–1. 0 mg/mL was used as a standard. The results are presented as milligrams of sinigrin equivalents per gram of dry weight (mg SinE/g dw) of the sample.

Oxalates were extracted with 70% (*v*/*v*) ethanol and measured at a wavelength of 520 nm, according to Naik [87]. An aqueous solution of oxalic acid in the range of 0.1875–1.5 mg/mL was used as a standard. The results are presented as milligrams per gram of dry weight (mg/g dw) of the sample.

Total phenolic compounds were determined in 70% (*v*/*v*) ethanol extracts using a method with the Folin–Ciocalteu reagent [88] at a wavelength of 740 nm. Gallic acid in the range of 0.1–1.7 mg/mL was used for the calibration curve. The results are presented as milligrams of gallic acid equivalents per gram of dry weight (mg GAE/g dw) of the sample.

Total flavonoids were measured at 520 nm, according to Zhishen et al. [89]. A quercetin solution in the range of 0.1–1.7 mg/mL was used to prepare the calibration curve. The results are presented as milligrams of quercetin equivalents per gram of dry weight (mg QE/g dw).

Total phenolic acids were analyzed according to a previously published method [90]. Absorbance was measured at a wavelength of 485 nm. A caffeic acid concentration in the range of 0.1–1.0 mg/mL was used to prepare the calibration line. The results are presented as milligrams of caffeic acid equivalents per gram of dry weight (mg CAE/g dw) of the sample.

Total hydroxycinnamic acids and flavonols were analyzed as in our previous work [91]. Absorbance was measured on a Nanodrop 2000c spectrophotometer at a wavelength of 320 nm for hydroxycinnamic acids and 355 nm for flavonols. A solution of caffeic acid in the range of 0.05–0.7 mg/mL for hydroxycinnamic acids and a solution of quercetin with a concentration of 0.00625–0.7 mg/mL for flavonols were used for the calibration curve. The results are presented as milligrams of caffeic acid equivalents per gram of dry weight (mg CAE/g dw) of the sample for hydroxycinnamic acids and as milligrams of quercetin equivalents per gram of dry weight (mg QE/g dw) of the sample for flavonols.

Total tannins were analyzed as in our previous work [83]. The absorbance was measured at a wavelength of 740 nm. Galic acid solutions with concentrations in the range of 0.05–1.3 mg/mL were used for the calibration line. The results are presented as milligrams of gallic acid equivalents per gram of dry weight (mg GAE/g dw) of the sample.

Total proanthocyanidins were analyzed as previously described [92]. The absorbance was measured at a wavelength of 485 nm. A cyanidin chloride solution with a concentration range of 0.05–0.5 mg/mL was used for the calibration curve. The results are presented as milligrams of cyanidin chloride equivalents per gram of dry weight (mg CcE/g dw) of the sample. For each of the methods, 70% ethanol was used instead of the extract as a blank. All these spectrophotometric measurements were conducted on an optical microplate reader Fluostar Optima (BMG LABTECH, Ortenberg, Germany).

Before the analysis of the individual compounds, ethanolic extracts were hydrolyzed using 1.2 M of HCl for 2 h under 80 °C and with shaking at 300 rpm. Separation, identification, and quantification of individual compounds were carried out on an Agilent 1100 Series device with a UV/VIS detector, non-polar Poroshell 120 SB-C18 column 4.6 × 75 mm with a particle size of 2.7 μm, and a pre-column Zorbax Rx-C18 4.6 × 12.5 mm with a particle size of 5 μm. The method of separation, identification, and quantification was as that reported in our previous work [93]. Flavonoids were analyzed at a wavelength of 360 nm, and phenolic acids at 310 nm. We identified the compounds by matching the retention times of the peaks in the extract analyses with those of standard reference compounds. Quantification was then performed using calibration curves derived from these standards.

### 3.6. Determination of Vitamins

For the analysis of the vitamins *L*-ascorbic and folic acid, extracts were hydrolyzed using 1.2 M of HCl, as previously described. Separation, identification, and quantification of vitamins were carried out on an Agilent 1100 Series device with a UV/VIS detector, and the conditions were as described in paragraph 3.5. Folic acid was analyzed at 280 nm, and *L*-ascorbic acid at 254 nm.

### 3.7. Determination of Oxidative Stress Parameters

Hydrogen peroxide (H_2_O_2_) content was determined as described in Junglee et al. [94]. The absorbance was quantified at 405 nm. Hydrogen peroxide content was calculated indirectly based on the calibration curve of standard H_2_O_2_ solutions of known concentrations (0.004–0.03 mg/mL). The results are expressed as mg of H_2_O_2_ per g of dw.

The proline content was evaluated using a method from Ljubej et al. [95]. The color intensity was quantified by measuring the absorbance at 520 nm. The proline content was calculated indirectly based on the calibration curve of standard *L*-proline solutions of known concentrations (0.02–0.6 mg/mL). The results are expressed as mg of *L*-proline per g of dw.

For the level of lipid peroxidation assessment, the method of Linić et al. [96] was used. Extracts were prepared in 0.1% trichloracetic acid. The absorbance was quantified at 532 and 600 nm. The malondialdehyde (MDA) content was calculated based on the molar extinction coefficient of 155 mM^−1^ cm^−1^, adapted for the measurement using the spectrophotometer (Thermo Scientific Nanodrop 2000c, Thermo Fisher Scientific, Waltham, MA, USA) and expressed as nmol of MDA per g of dry weight (dw).

### 3.8. Determination of Antioxidant Capacity

The antioxidant capacity was determined using three standard methods, namely ABTS, DPPH, and FRAP, adapted to small volumes, as already described [97]. The results are presented as percentage of inhibition or reduction (%) of the value obtained from a Trolox solution of the same concentration as our extracts.

### 3.9. GC-MS Analysis of Hormones

The extracts were prepared in isopropanol:acetic acid (95:5, *v*/*v*). To each sample, 100 ng of ^13^C_6_-IAA (Cambridge Isotope Laboratories, Andover, MA, USA) and 100 ng of D_6_-ABA (Cambridge Isotope Laboratories, Andover, MA, USA) was added as an internal standard. Methylation of the samples was carried out with tri-methylsilyldiazomethane [98]. The GC–MS analysis was carried out on a Varian Saturn 2100 ion-trap mass spectrometer using electron impact ionization at 70 eV, connected to a Varian CP-3900 gas chromatograph equipped with a CP-8400 autosampler (Varian, Walnut Creek, CA, USA), as described earlier [99]. The analyses were conducted using a Phenomenex ZB-5 column, 30 m × 0.25 mm × 0.25 m (Phenomenex, Darmstadt, Germany), using He carrier gas at 1 mL/min. The settings of the MS were as described in Campanella et al. [100]. The fragments analyzed for IAA were *m*/*z* 130/136, and, for ABA, *m*/*z* 190/194, endogenous/internal standard, respectively.

### 3.10. Extraction of Genomic and Plasmid DNA

The total genomic DNA from broccoli microgreens was isolated using CTAB [101] on 10 mg of lyophilized tissue. DNA concentrations were measured on a spectrometer at a wavelength of 260 nm.

Plasmid pSgM1_HNF1A was isolated from a bacterial overnight culture of *Escherichia coli* NEB^®^ Stable Competent *E. coli* (High Efficiency), which has spectinomycin selection, using the NucleoBond Xtra Maxi kit for transfection-grade plasmid DNA (Macherey-Nagel, Düren, Germany) commercial kit.

### 3.11. DNA Nicking Protection Assays

The DNA nicking protection assay was performed using the pSgM1_HNF1A (400 ng/µL) plasmid of 2854 bp in size which was mixed with plant extracts (30 mg dw/mL of 70% ethanol) and Fenton’s reagent (0.33 mM FeSO_4_ × 7 H_2_O, 0.323 M H_2_O_2_ in 1 × PBS, pH 3.5), according to Russo et al. [102]. Samples were incubated for 30 min at 37 °C. In the UV/H_2_O_2_ assay, the samples mixed with H_2_O_2_ and the plasmid were exposed to UV radiations for 6 min and 30 s [103].

Trolox, at a concentration of 30 mg/mL, was used as a positive control, while pure Fenton’s reagent, or H_2_O_2_ mixed with the plasmid, was used as a negative control. DNA samples were separated on 1.5% agarose gel in TBE buffer for 2 h and 50 min at 60 V and stained with GelRed^®^. The relative quantification of band density was conducted using the ImageJ (Fiji) software, version 2.2.0. For DNA mass calculation, we generated a standard curve by plotting the pixel density (*y*-axis) against the known DNA mass (*x*-axis), which was then used to quantify DNA in the samples. The obtained standard curve in the Fenton assay was described by the equation y = 1.2213x + 37.593, while, in the UV/H_2_O_2_ assay, it was y = 0.8283x + 35.317, where y represents pixel density and x represents the known DNA mass.

### 3.12. Statistical Analyses

Data were statistically processed using the Statistica 13.1 program (Stat Soft Inc., Krakow, Poland). A one-way analysis of variance (ANOVA), followed by Duncan’s new multiple range test (DNMRT) for multiple comparisons, was used to assess differences, with statistical significance set at *p* ≤ 0.05. A principal component analysis (PCA) was applied to illustrate the relationships between the samples and the measured parameters. To further visualize the groupings based on these parameters, hierarchical clustering (HC) was conducted using the Euclidean distance to gauge sample similarity or dissimilarity. Pearson’s correlation coefficients were calculated to analyze the relationships among parameters, with values of 0.60–0.79 indicating a high correlation and values of 0.80–1.00 indicating a very high correlation [50].

## 4. Conclusions

In total, we analyzed 40 variables. Drought significantly changed 68% of the variables, with 30% being increased and 38% being decreased (Figure S1). Flooding significantly changed 73% of the variables; 18% of these were increased and 55% were decreased. An opposite effect of flooding and drought was recorded for total carotenoids, glucosinolates, NO_3_^−^, oxalic acid, total phenolics, total phenolic acids, hydroxycinnamic acids, flavonols, proanthocyanidins, ferulic acid, sinapic acid, quercetin, kaempferol, *L*-ascorbic acid, folic acid, IAA, salicylic acid, ABTS, FRAP, H_2_O_2_, proline, and lipid peroxidation level. Therefore, we propose these variables as potential markers of drought and flooding. The most significant change that we recorded was for proline under the effect of drought, with proline increasing by 139% (Table 6, Figure S1).

Both drought and flooding reduced the amount of Chl *a*, and the effect of drought was more significant than the effect of flooding. Water stress was more detrimental for Chl *b* than Chl *a*, and flooding notably increased the Chl *a*/*b* ratio. Carotenoids were especifically affected: enhanced by drought and reduced by flooding. Total porphyrins decreased, with drought being significantly more effective than flooding. In general, drought showed a stronger negative effect than flooding on chlorophyll *a*, total chlorophyll, and total porphyrins. The photosynthetic parameters that were more susceptible to flooding than drought were total carotenoids. Total glucosinolates were differently affected by drought and flooding, as drought increased their content by 44% and flooding lowered it by 12%. This suggests that glucosinolates are more crucial for drought tolerance than for flood resilience. Drought increased the NO_3_^−^ content by 71%, while flooding decreased it by 61%. Oxalic acid was affected by drought only, and it was reduced by 5%. Flooding significantly reduced total proteins, flavonols, and tannins. Total phenolics were enhanced by drought and lowered by flooding. Total flavonoids were reduced by each stress type, with drought showing a stronger effect than flooding. Excessive water increased ferulic acid and quercetin; however, it reduced sinapic acid. Drought reduced ferulic acid but increased sinapic acid. The flavonol kaempferol was reduced by drought and enhanced by flooding. Based on these results, we suggest sinapic and ferulic acid and the flavonoid kaempferol as potential biomarkers for identifying and quantifying drought and flood stress in plants due to their distinct and consistent responses to varying water conditions. An analysis of the concentrations of these polyphenolic compounds might help in the development of targeted crop management strategies for specific water stresses. Each water stress type substantially altered H_2_O_2_ concentrations and, in an opposite way, drought increased the level of H_2_O_2_, while flooding decreased it. There was an enormous increase (139%) in proline under the effect of water shortage. In contrast, no effect was recorded in response to flooding. Flooding lowered the concentration of IAA and increased the hormone salicylic acid, while water shortage did not show an effect. Flooding caused a greater shift in antioxidant capacity of broccoli sprouts than drought. Both flooding and drought stress negatively affected broccoli genomic DNA concentration, with the drought effect being more significant. The drought-stressed broccoli extract was more effective in the preservation of supercoiled DNA forms exposed to the Fenton’s reagent than control and flood-stressed broccoli extracts. These findings underscore the importance of the stress type in shaping the protective potential of plant extracts, with implications for their application in antioxidant and DNA-protective contexts. The ability of drought-stressed broccoli extracts to maintain DNA in a supercoiled form highlights their potential as more effective DNA-protective agents, possibly useful in applications where DNA stability is crucial. However, since only one genotype of broccoli was tested, analyses of other genotypes are certainly necessary. Also, we suggest that future studies include F1 hybrids and landraces, both of which are known for their adaptability and resilience under diverse environmental conditions.

## Figures and Tables

**Table 1:** Effects of different watering regimes on photosynthetic pigments in broccoli sprouts.

mg/g dw	Control	Drought	Flooding	ΔD (%)	ΔF (%)
Chl *a*	3.96 ± 0.12 a	3.23 ± 0.16 c	3.49 ± 0.12 b	−18.44	−11.83
Chl *b*	2.20 ± 0.12 a	1.65 ± 0.08 b	1.70 ± 0.08 b	−24.78	−22.44
Chl *a* + Chl *b*	6.15 ± 0.20 a	4.88 ± 0.23 c	5.19 ± 0.19 b	−20.70	−15.61
Chl *+* Car	6.88 ± 0.22 a	5.68 ± 0.26 b	5.80 ± 0.20 b	−17.50	−15.68
Chl *a*/*b*	1.81 ± 0.09 b	1.95 ± 0.04 b	2.05 ± 0.06 a	8.23	13.54
Chl/(Chl + Car)	0.89 ± 0.00 a	0.86 ± 0.00 b	0.90 ± 0.00 a	−3.89	0.07
Car/(Chl + Car)	0.11 ± 0.00 b	0.14 ± 0.00 a	0.10 ± 0.00 b	33.00	−0.63
Chl/Car	8.50 ± 0.28 a	6.14 ± 0.08 b	8.55 ± 0.19 a	−27.79	0.65
Car	0.73 ± 0.03 b	0.80 ± 0.03 a	0.61 ± 0.02 c	9.71	−16.22
Por	11.45 ± 0.37 a	8.89 ± 0.40 c	9.69 ± 0.33 b	−22.38	−15.43

Values represent the average ± standard deviation of three biological and five technical replicates. Different letters indicate a significant difference among the values in a row (one-way ANOVA, Duncan’s test, *p* ≤ 0.05). Chl = chlorophyll; Car = carotenoids; D = drought; dw = dry weight; F = flooding; Por = porphyrins.

**Table 2:** Effects of different watering regimes on nitrates and proteins in broccoli sprouts.

	Control	Drought	Flooding	ΔD (%)	ΔF (%)
NO_3_^−^ (mg/g dw)	13.11 ± 1.05 b	22.41 ± 1.20 a	5.17 ± 1.03 c	70.99	−60.53
PROT (mg BSAE/g dw)	36.93 ± 1.83 a	34.80 ± 2.91 a	26.16 ± 2.32 b	−5.76	−29.17

Values represent the average ± standard deviation of three biological and three technical replicates. Different letters indicate a significant difference among the values in a row (one-way ANOVA, Duncan’s test, *p* ≤ 0.05). BSAE = bovine serum albumin equivalent; D = drought; dw = dry weight; F = flooding; NO_3_^−^ = total nitrates.

**Table 3:** Effects of different watering regimes on phytochemicals in broccoli sprouts.

	Control	Drought	Flooding	ΔD (%)	ΔF (%)
GLS (mg SinE/g dw)	26.67 ± 3.35 b	38.44 ± 2.26 a	23.59 ± 2.37 c	44.16	–11.53
OxA (mg/g dw)	48.94 ± 1.30 a	46.43 ± 0.64 b	50.01 ± 1.49 a	−5.14	2.19
TP (mg GAE/g dw)	12.37 ± 0.64 b	15.97 ± 0.53 a	10.53 ± 0.69 c	29.10	−14.84
TF (mg QE/g dw)	37.14 ± 2.49 a	28.58 ± 2.28 c	33.50 ± 2.13 b	−23.03	−9.80
TPA (mg CAE/g dw)	10.94 ± 0.47 ab	11.27 ± 0.63 a	10.68 ± 0.29 b	3.08	−2.38
THCA (mg CAE/g dw)	11.59 ± 1.01 a	12.74 ± 1.78 a	10.13 ± 1.12 b	9.89	−12.65
TFlo (mg QE/g dw)	18.18 ± 1.29 a	18.80 ± 2.81 a	16.12 ± 1.67 b	3.38	−11.37
TT (mg GAE/g dw)	7.54 ± 1.12 a	7.50 ± 0.83 a	4.84 ± 0.97 b	−0.58	−35.85
TPAN (mg CcE/g dw)	2.62 ± 0.16 b	2.80 ± 0.23 a	2.12 ± 0.16 c	6.91	−18.92

Values represent the average ± standard deviation of three biological and three technical replicates. Different letters indicate a significant difference among the values in a row (one-way ANOVA, Duncan’s test, *p* ≤ 0.05). D = drought; dw = dry weight; GLS = total intact glucosinolates; SinE = sinigrin equivalent; OxA = oxalic acid; CAE = caffeic acid equivalent; CcE = cyanidin chloride equivalent; F = flooding; GAE = gallic acid equivalent; QE = quercetin equivalent; TF = total flavonoids; TFlo = total flavonols; THCA = total hydroxycinnamic acids; TP = total phenolics; TPA = total phenolic acids; TPAN = total proanthocyanidins; TT = total tannins.

**Table 4:** Effects of different regimes of watering on individual phenolic compounds in broccoli sprouts.

mg/g dw	Control	Drought	Flooding	ΔD (%)	ΔF(%)
Ferulic acid	0.16 ± 0.01 b	0.13 ± 0.00 c	0.25 ± 0.01 a	−16.91	56.29
Sinapic acid	2.04 ± 0.03 b	2.65 ± 0.12 a	1.77 ± 0.02 c	29.77	−13.13
Kaempferol	0.71 ± 0.03 b	0.58 ± 0.04 c	0.87 ± 0.06 a	−18.13	22.29
Quercetin	0.18 ± 0.01 b	0.16 ± 0.02 b	0.25 ± 0.02 a	−12.10	37.49

Values represent the average ± standard deviation of three biological replicates. Different letters indicate a significant difference among the values in a row (one-way ANOVA, Duncan’s test, *p* ≤ 0.05). D = drought; dw = dry weight; F = flooding.

**Table 5:** Effects of different watering regimes on vitamins in broccoli microgreens.

mg/g dw	Control	Drought	Flooding	ΔD (%)	ΔF (%)
*L*-ascorbic acid	1.48 ± 0.10 a	1.48 ± 0.10 a	1.39 ± 0.06 a	0.25	−5.88
Folic acid	5.37 ± 1.12 a	5.38 ± 0.98 a	4.62 ± 1.13 a	0.06	−14.10

Values represent the average ± standard deviation of three biological replicates. Different letters indicate a significant difference among the values in a row (one-way ANOVA, Duncan’s test, *p* ≤ 0.05). D = drought; dw = dry weight; F = flooding.

**Table 6:** Effects of different watering regimes on oxidative stress parameters in broccoli microgreens.

	Control	Drought	Flooding	ΔD (%)	ΔF (%)
H_2_O_2_ (mg/g dw)	0.32 ± 0.05 b	0.41 ± 0.03 a	0.26 ± 0.02 c	25.77	−20.58
Proline (mg/g dw)	6.65 ± 1.28 b	15.88 ± 0.67 a	6.17 ± 0.44 b	138.84	−7.20
Lipid peroxidation (nmol MDA/g dw)	42.87 ± 0.69 b	42.29 ± 0.79 c	53.89 ± 0.98 a	−1.34	25.71

Values represent the average ± standard deviation of three biological replicates. Different letters indicate a significant difference among the values in a row (one-way ANOVA, Duncan’s test, *p* ≤ 0.05). D = drought; dw = dry weight; F = flooding; MDA = malondialdehyde.

**Table 7:** Effects of different watering regimes on antioxidant capacity of broccoli microgreens extracts.

	Control	Drought	Flooding	ΔD (%)	ΔF (%)
ABTS (% inhibition)	72.22 ± 2.49 b	78.34 ± 3.34 a	59.07 ± 3.24 c	8.48	−18.21
DPPH (% inhibition)	61.40 ± 5.86 c	71.05 ± 5.00 b	87.57 ± 3.88 a	15.71	42.62
FRAP (% reduction)	87.92 ± 0.74 a	88.32 ± 0.68 a	86.72 ± 0.36 b	0.45	−1.36

Values represent the average ± standard deviation of three biological and three technical replicates. Different letters indicate a significant difference among the values in a row (one-way ANOVA, Duncan’s test, *p* ≤ 0.05). D = drought; F = flooding.

**Table 8:** Effects of different watering regimes on the concentration of hormones in broccoli microgreens.

µg/g dw	Control	Drought	Flooding	ΔD (%)	ΔF (%)
ABA	66.57 ± 28.70 a	31.75 ± 15.29 a	42.15 ± 23.93 a	−52.30	−36.69
IAA	440.01 ± 156.82 a	437.28 ± 88.19 a	98.74 ± 27.89 b	−0.62	−77.56
Salicylic acid	140.83 ± 3.18 b	128.61 ± 18.22 b	260.97 ± 9.54 a	−8.68	85.31

Values represent the average ± standard deviation of three biological replicates. Different letters indicate a significant difference among the values in a row (one-way ANOVA, Duncan’s test, *p* ≤ 0.05). D = drought; dw = dry weight; F = flooding.

## Data Availability

The data that support the findings of this study are available from the corresponding author I.Š., upon request.

## Supplementary Materials

The following supporting information can be downloaded at: https://www.mdpi.com/article/10.3390/ijms26020632/s1.
