# Supplementary figures and images for: Impact of Water Stress on Metabolic Intermediates and Regulators in Broccoli Sprouts, and Cellular Defense Potential of Their Extracts
Source: Int J Mol Sci. 2025 Jan 13;26(2):632. doi: 10.3390/ijms26020632 (PMC11765553; doi:10.3390/ijms26020632)

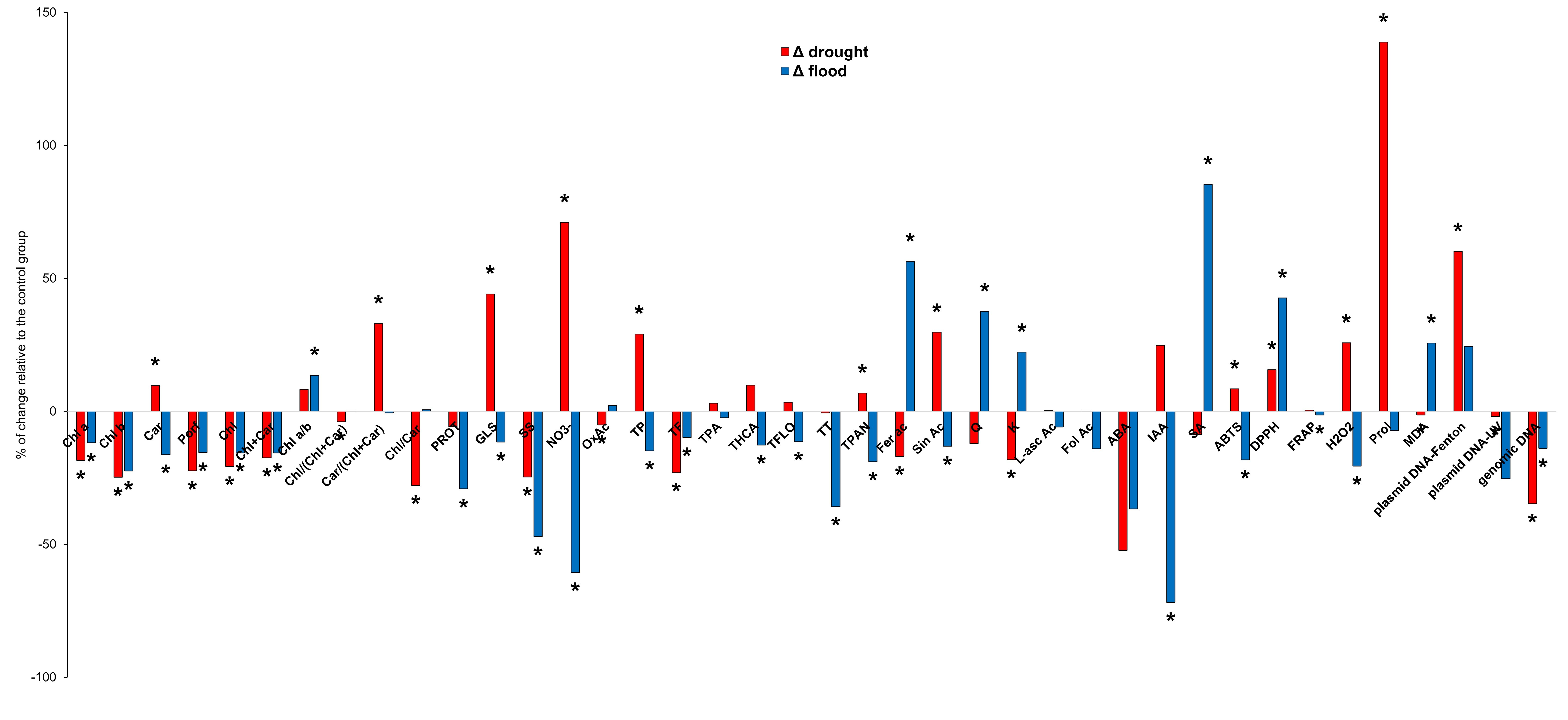

Supplement: Supplementary file 1 [file ijms-26-00632-s001.zip › Figure S1.jpg]
